# A Novel Anti-Kv10.1 Nanobody Fused to Single-Chain TRAIL Enhances Apoptosis Induction in Cancer Cells

**DOI:** 10.3389/fphar.2020.00686

**Published:** 2020-05-14

**Authors:** Franziska Hartung, Thomas Krüwel, Xiaoyi Shi, Klaus Pfizenmaier, Roland Kontermann, Patrick Chames, Frauke Alves, Luis A. Pardo

**Affiliations:** ^1^Oncophysiology Group, Max Planck, Institute of Experimental Medicine, Göttingen, Germany; ^2^Institute of Diagnostic and Interventional Radiology, University Medical Center Göttingen, Göttingen, Germany; ^3^Institut für Zellbiologie und Immunologie, Universität Stuttgart, Stuttgart, Germany; ^4^Aix Marseille Univ, CNRS, Inserm, Institut Paoli-Calmettes, CRCM, Marseille, France; ^5^Translational Molecular Imaging Group, Max Planck Institute of Experimental Medicine, Göttingen, Germany

**Keywords:** targeted therapy, nanobody, TRAIL, Kv10.1, voltage-gated potassium channel, apoptosis

## Abstract

Antibody-based therapies hold promise for a safe and efficient treatment of cancer. The identification of target tumor cells through a specific antigen enriched on their surface and the subsequent delivery of the therapeutic agent only to those cells requires, besides the efficacy of the therapeutic agent itself, the identification of an antigen enriched on the surface of tumor cells, the generation of high affinity antibodies against that antigen. We have generated single-domain antibodies (nanobodies) against the voltage-gated potassium channel Kv10.1, which outside of the brain is detectable almost exclusively in tumor cells. The nanobody with highest affinity was fused to an improved form of the tumor necrosis factor-related apoptosis inducing ligand TRAIL, to target this cytokine to the surface of tumor cells. The resulting construct, VHH-D9-scTRAIL, shows rapid and strong apoptosis induction in different tumor models in cell culture. The construct combines two sources of specificity, the expression of the antigen restricted to tumor cells and the tumor selectivity of TRAIL. Such specificity combined with the high affinity obtained through nanobodies make the novel agent a promising concept for cancer therapy.

## Introduction

Antibody engineering is an essential process to improve the efficacy of antibody-based therapeutics. Beside the whole monoclonal antibody (mAb) formats with their two antigen-binding arms and Fc-region, which can be modified and gain diverse effector functions, small antibody fragments, like single-chain fragments (scFv), diabodies, or nanobodies are in the focus of innovative therapeutic and diagnostic strategies. Nanobodies, the variable domain of (VHH) heavy-chain antibodies from camelids, play an essential role in this context, because of their particular stability and small size (15 kDa) (Muyldermans, 2013; Chanier and Chames, 2019). The VHH domain consists of four framework regions (FR) and three highly variable loops, the complementarity determining regions (CDR), which form the antigen-binding interface (paratope) and determine the nanobody specificity. Despite the reduced size of their paratope, nanobodies can bind their target antigen with affinities comparable to those observed in mAbs (Muyldermans et al., 2001).

In this study we describe the design of a nanobody-based affinity protein targeting the tumor-associated antigen Kv10.1, a voltage-gated potassium channel overexpressed in different tumor tissues and many cancer cell lines (Pardo and Stühmer, 2014; Ouadid-Ahidouch et al., 2016) and references therein). We have been interested for many years in the use of voltage-gated potassium channels, and specifically Kv10.1, as therapeutic targets in cancer. Outside of the brain, Kv10.1 is shortly expressed at the end of the cell cycle, where it participates in the finalization of mitosis. Therefore, at any given time, very few non-neuronal cells express the channel on their surface (Sánchez et al., 2016; Urrego et al., 2016). This time-dependent expression pattern is tightly controlled, and therefore Kv10.1 is at the crossroads of many transcription factors and non-coding RNAs (e.g., (Lin et al., 2011; Bai et al., 2013; Wu et al., 2013; Sánchez et al., 2016; Urrego et al., 2016; Horst et al., 2017; Hsu et al., 2017)). The complex regulation is often lost in tumors, and Kv10.1 becomes abundantly expressed in a majority of human cancers (e.g., (Hemmerlein et al., 2006; Ding et al., 2007; Spohr et al., 2007; Ding et al., 2008; Menendez et al., 2012; Lai et al., 2014)), indicating that its function is beneficial for the cancer cell. Inhibition of channel function has been proposed as a therapeutic strategy (Gómez-Varela et al., 2007; Downie et al., 2008; De Guadalupe Chavez-Lopez et al., 2015), and there is evidence that this approach can prolong survival in patients with brain metastases (Martínez et al., 2015). Alternatively, the channel can be used as a marker for the tumor cell due to its scarce expression in normal tissues outside of the brain (Hartung and Pardo, 2016). Based on structural analysis of our already developed mouse-derived scFv antibody (Gómez-Varela et al., 2007) and the newly isolated anti-Kv10.1 nanobodies, we generated a new fusion construct with the apoptosis-inducing ligand TRAIL (Tumor Necrosis Factor-Related Apoptosis-Inducing Ligand) (Wiley et al., 1995).

TRAIL is an attractive candidate for cancer treatment and for the design of antibody-fusion constructs since it has been demonstrated that it is able to selectively induce apoptosis in cancer (Ashkenazi et al., 1999). TRAIL is expressed on the surface of immune cells and binds to five different receptors. Two of them, TRAIL-R1 and TRAIL-R2, induce caspase activation and apoptotic cell death after ligand binding. TRAIL-R3 and TRAIL-R4 are decoy receptors, also expressed on the cell surface but lacking functional intracellular death domains. Membrane-bound TRAIL (on the membrane of immune cells) shows increased ability to induce receptor clustering and more efficient activation of the apoptotic signaling (Muhlenbeck et al., 2000; Von Karstedt et al., 2017), and this can be mimicked by functionalization with agents binding to surface antigens (Wajant et al., 2001). Conversely, soluble TRAIL has also been shown to induce an inflammatory response that can result in higher migration of the tumor cells and thereby tumor spread (Zhou DH, 2013; von Karstedt et al., 2015; Hartwig et al., 2017). Active targeting of TRAIL to cancer cells by fusion with an antibody moiety targeting a tumor-associated antigen enriched in tumor cells results not only in increased accumulation of the fusion proteins at the tumor site, but also mimics membrane-bound presentation of TRAIL, thus allowing higher efficacy and less inflammatory response. We and others have already shown the improved activity of antibody-TRAIL fusion constructs (Stieglmaier et al., 2007; Bremer et al., 2008; Hartung et al., 2011; Hartung and Pardo, 2016).

The homotrimeric structure of active TRAIL can lead to decreased therapeutic efficiency by dissociation into the monomeric subunits. To overcome this drawback, in this study we used the single-chain variant of TRAIL (scTRAIL), a fusion of three single TRAIL fragments *via* short peptide linkers that shows enhanced apoptosis induction (Seifert et al., 2014; Hutt et al., 2018; Siegemund et al., 2018). The properties of nanobodies (small size, high stability and solubility, high affinity (Jovcevska and Muyldermans, 2020) have been already used in combination with TRAIL. Nanobodies against EGFR fused to TRAIL have shown efficacy against tumor cells resistant to both strategies (inhibition of EGFR and activation of TRAIL) when used separately (Zhu et al., 2017).

In this study, we describe a high affinity construct, VHH-D9-scTRAIL, that targets a TRAIL variant with enhanced proapoptotic activity to tumor cells in cell culture models. The construct combines the specificity of Kv10.1 as tumor-associated antigen with the small size and high stability of nanobodies and the efficacy of scTRAIL as a promising candidate to overcome resistance to conventional chemotherapy.

## Results

### Generation of Anti-Kv10.1 VHH Nanobodies

**Figure 1:**
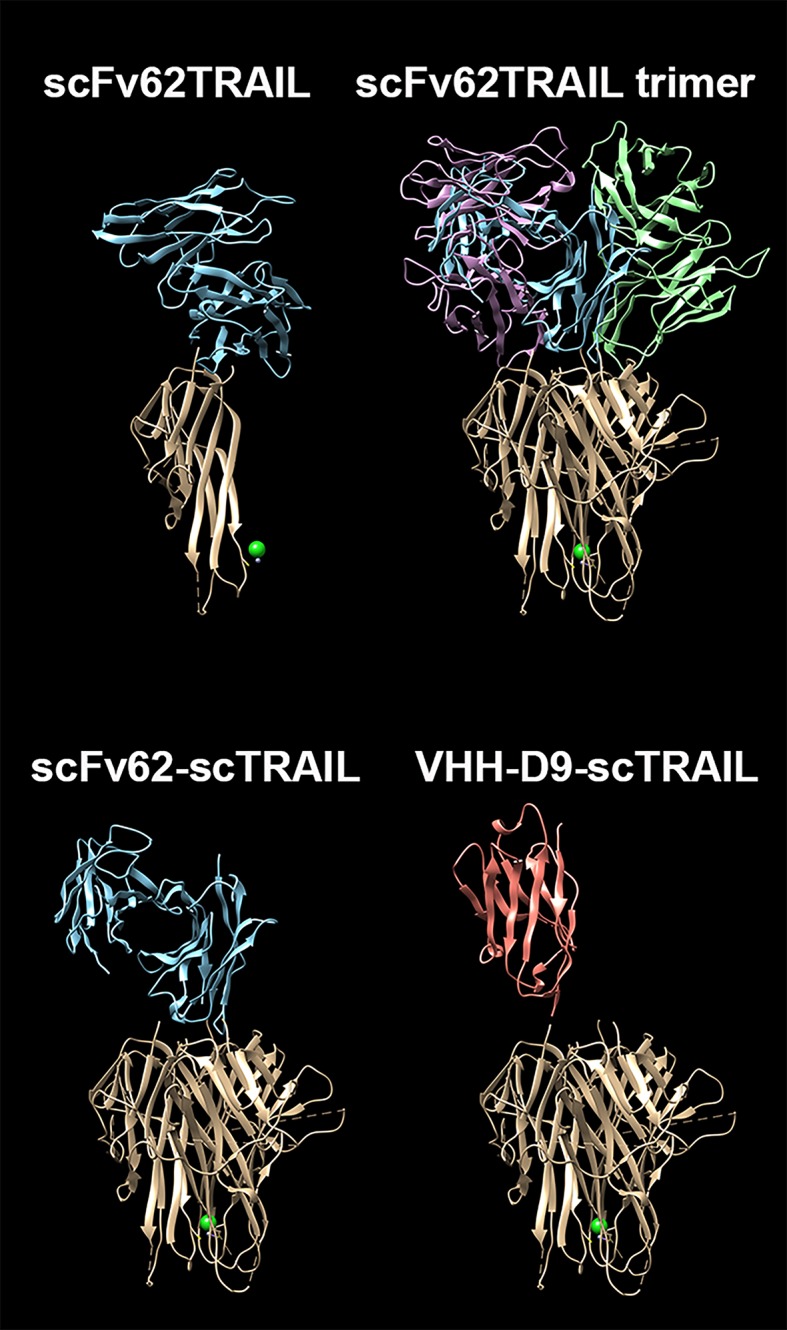
Sequence alignment of the anti-Kv10.1 nanobodies. CDR1, 2, and 3 are underlined. Residues are colored according to chemistry. Note the strong similarity between C4 and D9, especially the conserved CDR2 and the short CR3.

Anti-Kv10.1 nanobodies were generated by immunization of a llama with a Kv10.1-derived antigen, already successful in generating mouse anti-Kv10.1 mouse mAb (Hemmerlein et al., 2006). The antigen encompasses the E3 segment of the channel, which corresponds to the extracellular linker between S5 and S6 transmembrane segments and is remarkably long in this channel family, and extends to the pore loop. With the aim to induce tetramerization of the target sequences, E3 was fused to the C-terminal tetramerizing coiled-coil of the channel (Jenke et al., 2003). The resulting antibody response is therefore likely to target the extracellular (exposed) domains. The construct contains also thioredoxin (TRX) to increase solubility and stability (Lavallie et al., 1993). **Figure S1** shows a schematic view of the antigen and its conservation among mammalian species. After immunization, the resulting phage display library of 1.3x10^7^ clones was rescued within the helper phage KM13 and enriched through 9 rounds of depletion on immobilized TRX and incubation with different concentrations of the antigen. 186 clones where then screened on the antigen and negatively on TRX, resulting in 30 hits. Of those, ten clones were amplified and induced for nanobody production. Sequencing revealed nine independent clones (**Figure 1**). A detailed look to the primary structure of those binders revealed clustering into two classes, with pronounced differences in complementarity determining regions (CDR) 2 and 3. Nanobodies VHH-C4 and VHH-D9 shared a remarkably short CDR 3 and therefore had a lower molecular weight compared to all other tested clones (**Figure S2**). CDR1 was relatively conserved in all antibodies, and the framework (FR) regions were conserved throughout all positive binders.

**Figure 2:**
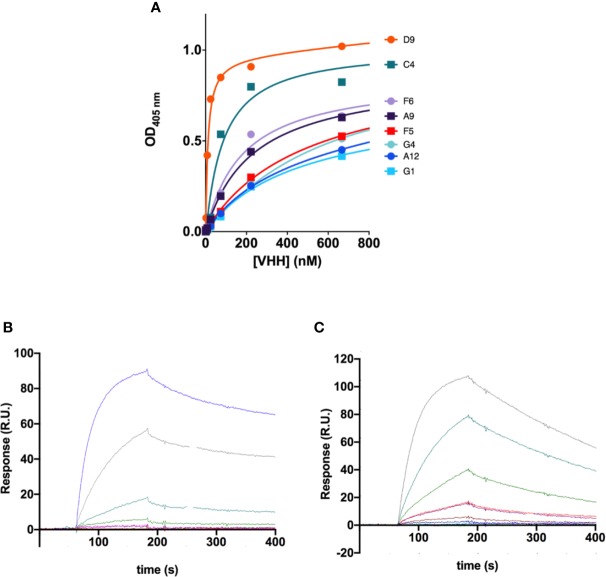
Affinity of the anti-Kv10.1 nanobodies. **(A)** Apparent affinities of the VHH antibodies determined by ELISA against the antigen used for immunization. The values resulting from the fit are listed in **Table 1**. D9 and C4 showed the highest affinity. **(B)** Affinity and kinetics of binding of VHH C4 on h1x immobilized on a surface plasmon resonance (SPR) sensor chip. The concentration used in each sesorgram is listed in the main text. R.U.: resonance units. **(C)** SPR sensorgrams for nanobody D9.

We next estimated the apparent affinities of the VHH antibodies for the recombinant antigen by ELISA (**Figure 2A**). All nanobodies showed affinities in the submicromolar range, but the two clones with shorter CDR3 (C4 and D9) had clearly higher apparent affinities (K_d_ of 81 nM and 11 nM respectively; **Table 1**, **Figure 2A**). It is worth pointing out that those two antibodies share sequence homology with the mouse monoclonal antibody showing highest affinity against this region in our previous studies (mAb62), which was the starting sequence for our single chain antibody scFv62 (Hartung et al., 2011).

**Table 1:** Apparent affinity of the nanobodies determined by ELISA.

Nanobody	A9	A12	C4	D9	F5	F6	G1	G4
K_d_ (nM)	223	343	87	11	470	177	431	943

The affinity of these two antibodies was also assessed by surface plasmon resonance (SPR) to determine affinity (K_D_), association (k_a_), and dissociation (k_d_) constants. A serial dilution of nanobodies D9 (3.12 nM to 200 nM, **Figure 2B**) and C4 (25 nM to 400 nM, **Figure 2C**) was flown as analyte over the recombinant antigen used for immunization immobilized on the SPR chip. Affinities of 1.8 μM and 78 nM for C4 and D9, respectively, were determined by using a 1:1 Langmuir model resulting in comparable K_D_ values as previously determined for D9. Interestingly, C4 is characterized by both, a slower association and a slower dissociation compared to D9.

### Construction of VHH-D9-scTRAIL Fusions

**Figure 3:**
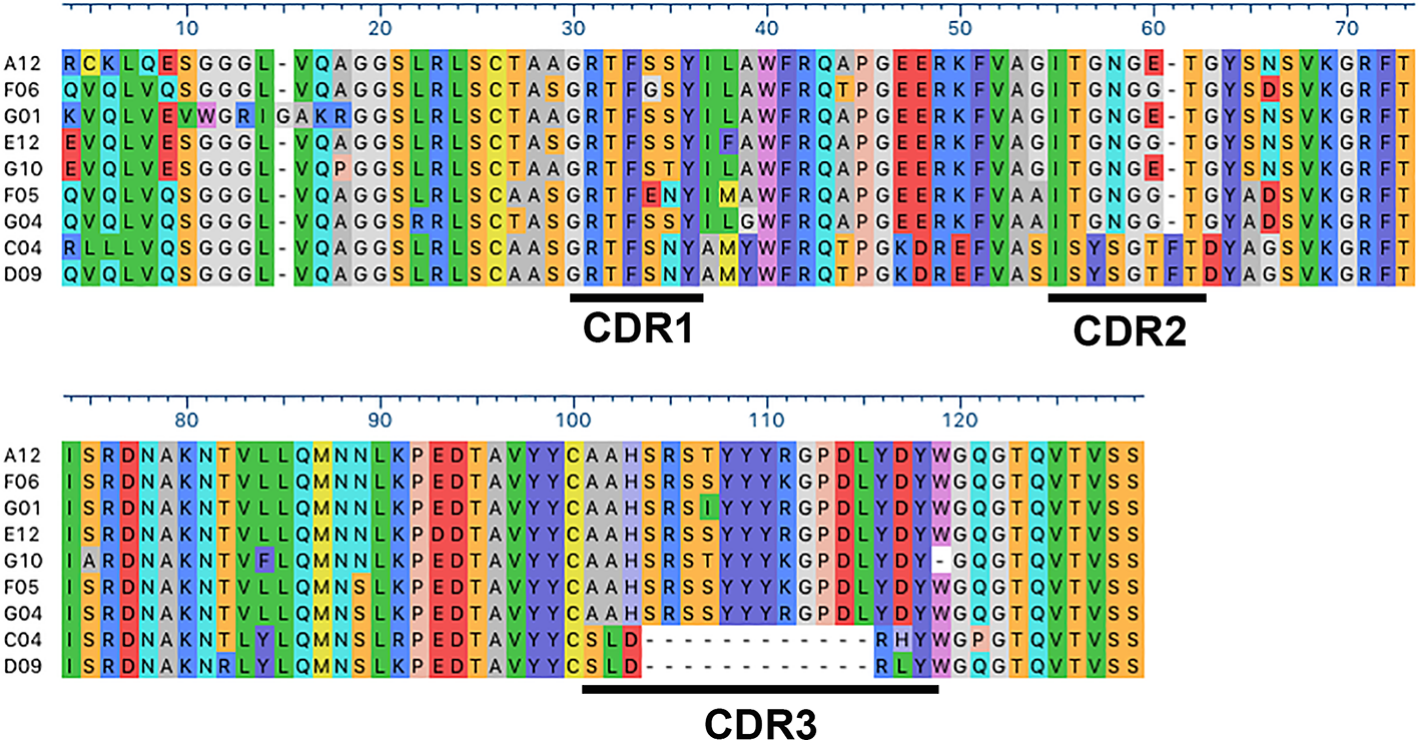
Comparison of the predicted structures of the constructs. The three-dimensional structure of scFv62 as a monomer, its assembly as a functional trimer, and the three-dimensional (3-D) structures of scFv62-scTRAIL and VHH-D9-scTRAIL are depicted with the different moieties in different colors (blue, green and magenta for scFv62, red for VHH and light brown for TRAIL). The TRAIL trimer structure corresponds to PDB ID 1dg6 (Hymowitz et al., 2000). The structures of scFv62 and VHH-D9 have been modelled using AbodyBuilder (Leem et al., 2016). The PDB files can be found in Supplementary Information.

Given its superior binding properties, we selected the nanobody D9 for subsequent studies. Our model for the design of a targeted therapy against Kv10.1 includes proteins fused to TRAIL, which have shown efficacy *in vitro* and *in vivo*. Since single chain TRAIL (scTRAIL, a tandem repeat of three TRAIL subunits to maintain trimeric structure) is superior in inducing apoptosis to monomeric TRAIL (Siegemund et al., 2018), we decided to use this strategy for the VHH fusion. scTRAIL was also fused to our previously reported scFv62 antibody (Hartung et al., 2011) to compare the behavior of the two types of antibodies. **Figure 3** shows schematically the structural differences of our validated scFv62-TRAIL (Hartung et al., 2011) and the new scTRAIL fusions. The constructs were produced in CHO-K1 cells using the pSecTag system to get the product secreted into the medium.

### VHH-D9-scTRAIL Maintains Affinity and Specificity

**Figure 4:**
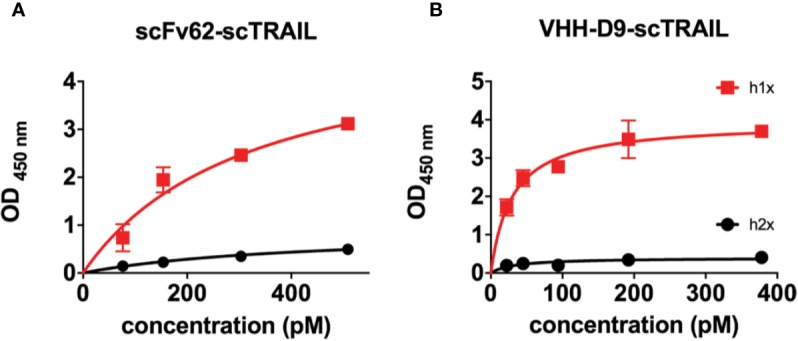
Apparent affinity and selectivity of scFv62-scTRAIL and VHH-D9-scTRAIL. h1x and h2x antigens were immobilized on ELISA plates and incubated with different concentrations of the relevant fusion proteins. Binding of the fusion was measured by detection of TRAIL. Results on scFv62-scTRAIL are shown in **(A)**, and those of VHH-D9-scTRAIL in panel **(B)**. Black line and symbols correspond to h2x, which produced weak signals in both cases. Red color indicates data obtained with h1x. Error bars indicate standard deviation.

We first checked whether fusion of the antibodies to scTRAIL impairs the affinities and/or the specificities of the antibodies. The two scTRAIL fusions, scFv62-scTRAIL and VHH-D9-scTRAIL were analyzed for their binding affinity to the recombinant antigen h1x, which is the same protein used to rise both antibodies. We also tested affinity for a similar protein derived from the closely related channel Kv10.2, that is, the E3 domain of Kv10.2 and its tetramerizing coiled-coil (h2x). As shown in **Figure 4**, the apparent affinity to h1x and h2x was assessed by ELISA. Plates were coated with the antigen (h1x or h2x) and a commercial TRAIL ELISA kit was used for detection. It is unclear how accurate the absolute values for apparent affinity are because of the stable trimeric structure of TRAIL in the construct, but the relative values should be informative as to how scFv and VHH compare to each other. The apparent affinities of scFv62-scTRAIL and VHH-D9-scTRAIL constructs measured in these experiments were 280 pM and 29 pM, respectively. The large difference observed between the apparent affinity of VHH-D9-scTRAIL and of VHH-D9 alone may be explained by the different methods used to detect the nanobody alone (His-Tag, one epitope per antibody molecule) and the fusion construct (TRAIL, which is trivalent). VHH-D9-scTRAIL showed a 10-fold higher apparent affinity to the antigen than scFv62-scTRAIL. Therefore, both constructs retain affinity for h1x. Both also maintain specificity, because none of the constructs showed binding to h2x (**Figure 4**, black symbols).

### VHH-D9-scTRAIL Induces Apoptosis in Kv10.1 Positive Tumor Cells

**Figure 5:**
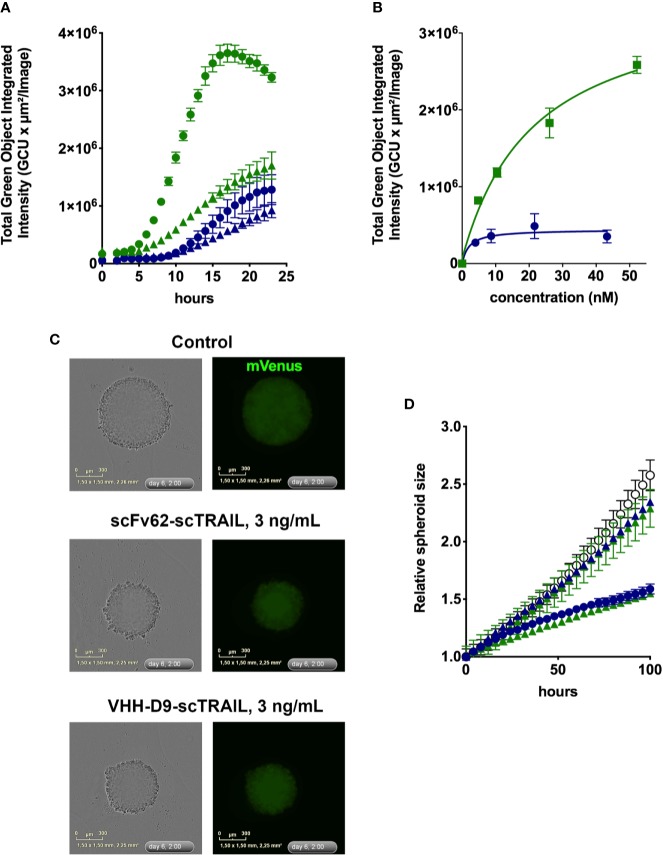
Apoptosis induction by scFv62-scTRAIL and VHH-D9-scTRAIL on DU-145 cells. **(A)** Cells seeded in 96 well plates were treated with 370 pg/ml (triangles) or 3.7 ng/ml (circles) of scFv62-scTRAIL (corresponding to 4.3 pM and 43 pM, blue) or VHH-D9-scTRAIL (corresponding to 5.2 pM and 52 pM, green), and apoptosis was measured over time using a green fluorescent caspase reporter. All conditions induced apoptosis, but especially VHH-D9-scTRAIL produced a large fraction of apoptotic cells as early as 8 h after the start of treatment. **(B)** Dose-response of the apoptosis induction by scFv62-scTRAIL (blue) and VHH-D9-scTRAIL (green). **(C)** Representative images of spheroids produced by fluorescent (mVenus) DU-145 cells and submitted to the indicated treatments. **(D)** Relative size of the spheroids treated with the fusion constructs at 300 pg/ml and 3 ng/ml (corresponding to 4.2 and 42 pM for VHH-D9-scTRAIL and 3.5 and 35 pM for scFV62-scTRAIL). The values in response to lower doses of the constructs did not differ from the control (open black circles), while higher doses reduced spheroid size for both scFv62-scTRAIL (blue) and VHH-D9-scTRAIL (green). All data presented as means ± SD.

In the next step, we studied the efficacy of the VHH-D9-scTRAIL construct in inducing apoptosis in Kv10.1-expressing human prostate cancer cells (DU-145) in conventional cell culture. To analyze the process of apoptosis induction we used the live-cell imaging system IncuCyte and a caspase assay to quantify apoptosis induction over time. Activation of caspase 3/7 is reported as an increase in fluorescence (**Figure 5A**).

Due to its low toxicity in the time window tested, cycloheximide (CHX) was used subsequently as sensitizer for all *in vitro* experiments. **Figure 5A** shows the development of the apoptotic signal in DU-145 cells over time after treatment with different concentrations of the two scTRAIL fusion constructs over time. Comparison of the apoptotic signals in response to scFv62-scTRAIL and VHH-D9-scTRAIL shows more efficient and faster apoptosis induction by VHH-D9-scTRAIL starting after 4 h of treatment. Analysis of the apoptotic signals at 12 h after treatment and different concentrations of both scTRAIL constructs illustrates the clear difference in the intensity of apoptosis induction between them, three times more in the case of VHH-D9-scTRAIL, although its EC50 (~20 nM) was higher than that of scFv62-scTRAIL (~2 nM; **Figure 5B**).

In terms of response to therapy, it is generally accepted that three-dimensional (3-D) cultures are more similar to the real tumors than 2-D cultures. Because a relevant difference between the two constructs is size, likely affecting the ability to penetrate the tumor, we set out to test efficacy of the constructs on the growth and viability of tumor spheroids. We used a DU-145-derived cell line that expresses constitutively the fluorescence reporter protein mVenus (Hartung et al., 2011). We measured over time the change in size of the spheroids by determining the surface occupied by green fluorescence by live cell imaging in the IncuCyte system (**Figure 5C**). Both constructs inhibited the growth of the spheroids with similar efficacy (**Figure 5D**); the spheroids grew one-half of the controls during the time of the experiment in the presence of higher doses of scTRAIL constructs (3 ng/ml).

The lack of a difference in efficacy between both fusion constructs was surprising, because VHH-D9-scTRAIL was more potent than scFv62-scTRAIL in 2-D cultures, and it is also expected to have improved tumor penetration and higher affinity, and thereby a stronger effect in spheroids. We hypothesized that this could be due to the fast growth of DU-145 spheroids. Therefore, we performed similar experiments using the pancreatic cancer cell line Capan-1, which on one hand shows slower growth, and on the other hand is strongly influenced by co-culture with stromal cells.

**Figure 6:**
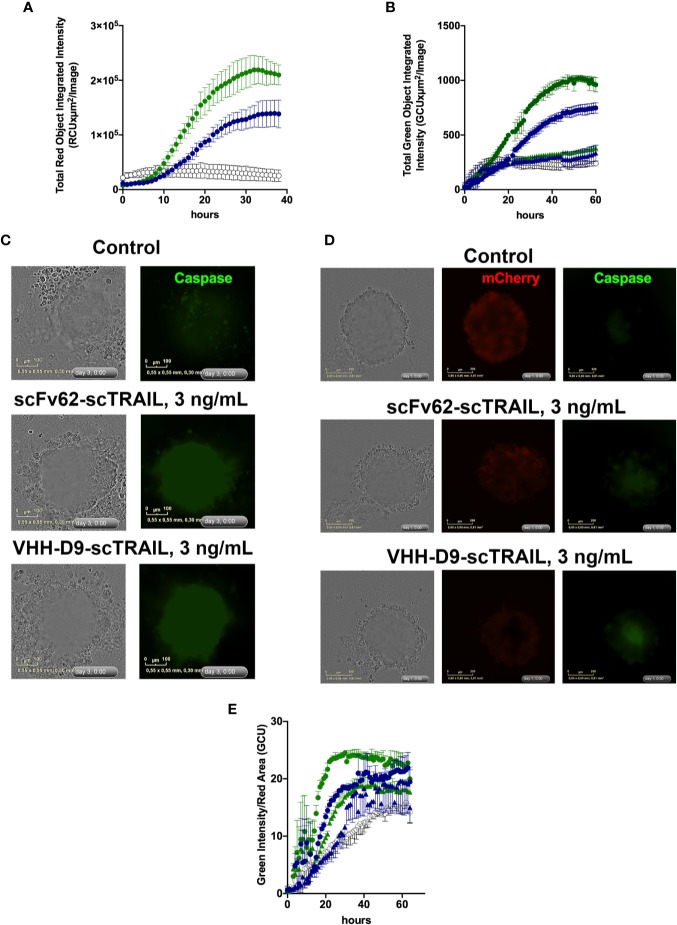
Apoptosis induction by scFv62-scTRAIL and VHH-D9-scTRAIL on Capan-1 cells. **(A)** Cells seeded in 96 well plates were treated with 3 ng/ml of scFv62-scTRAIL (35 pM, blue) or VHH-D9-scTRAIL (42 pM, green), and apoptosis was measured over time using a green fluorescent caspase reporter. VHH-D9-scTRAIL produced a larger fraction of apoptotic cells. **(B)** Apoptosis induction in Capan-1 spheroids of 0.3 ng/ml (triangles) or 3 ng/ml (circles) VHH-D9-scTRAIL (green) or scFv62-scTRAIL. The effects were similar to those observed in two-dimensional (2-D) culture, albeit delayed in time. **(C)** Representative images of spheroids produced by Capan-1 cells and treated as indicated. Green fluorescence indicates caspase 3/7 activation. **(D)** Representative images of spheroids produced by Capan-1 cells co-cultured with stellate cells and treated as indicated. Red fluorescence identifies stellate cells (mCherry), while green corresponds to caspase activation. **(E)** Apoptosis induction in Capan-1 spheroids cocultured with RLT-PSCs expressing mCherry relative to the size of the compact spheroid. Apoptosis was detected as green fluorescence, and the size of the compact spheroid was inferred from the area of red fluorescence from stellate cells. There was fast and intense induction of apoptosis when 3 ng/ml (circles) VHH-D9-scTRAIL (green) or scFv62-scTRAIL were added to the medium. 0.3 ng/ml (triangles) showed little apoptosis induction. All data presented as means ± SD.

In standard 2-D culture, both VHH-D9-scTRAIL and scFv62-scTRAIL effectively induced apoptosis in the presence of CHX in Capan-1 cells. As expected, VHH-D9-scTRAIL had a more intense effect, corresponding to its higher affinity to Kv10.1 (**Figure 6A**). When grown as single culture, spheroids of Capan-1 cells showed remarkable apoptosis in the presence of both scTRAIL constructs, both in quantitative terms (**Figure 6B**) and morphologically (**Figure 6C** and **Video S1**). VHH-D9-scTRAIL was again remarkably more efficacious than scFv62-scTRAIL inducing apoptosis in this model. The effect was even more evident when the spheroids were formed by a mixture of Capan-1 and stellate cells (RLT-PSCs (Jesnowski et al., 2005); 4,000 cells of each type at the time of seeding). In order to distinguish between tumor and stellate cells, RLT-PSC were stably transfected with the red fluorescent reporter mCherry protein. Live imaging of the spheroids (**Figure 6D**, **Video S1**) revealed that the stellate cells were not affected by the presence of the constructs, but the apoptosis induction was fast and intense in the tumor cells, measured as green fluorescence originated by cleavage of caspase 3/7 substrate, with apoptosis already observed 12 h after the start of treatment (**Figure 6E**). We used the surface occupied by red fluorescence from the RLT-PSC to normalize for the size of the spheroid. The lower dose of VHH-D9-scTRAIL used (300 pg/mL) was similarly efficacious as the higher dose of scFv62-scTRAIL (3 ng/ml).

## Discussion

In this work, we have combined the specificity for tumor cells and accessibility of Kv10.1 with the advantages of nanobodies and of scTRAIL to generate a fusion construct with enhanced apoptosis inducing activity targeting different tumor cells.

The use of scTRAIL, besides enhanced efficacy, leads to size reduction, since the construct contains just only one antibody unit instead of three; however, it also requires higher affinity antibodies. scTRAIL has been shown to bind with affinity of approximately 1 nM to the TRAIL-R2 receptor; therefore, to take advantage of binding to the tumor antigen, the affinity of the antibody needs to be higher (Hutt et al., 2018). Therefore, to improve antibody affinity, we generated a series of single domain antibodies, which can achieve very high binding affinity combined with smaller size and better stability (Xenaki et al., 2017; Chanier and Chames, 2019). Two of the isolated VHHs showed high apparent binding affinity, in one case (D9) in the low nM range. Interestingly, the two nanobodies with highest apparent affinity show a remarkably short CDR3, with just six residues. Comparative analysis of 90 different nanobody sequences identifies a mean CDR3 length of 15 residues (Mitchell and Colwell, 2018), which also holds true for most of the VHHs identified in our screening.

We show that the two constructs carrying scTRAIL, scFv62-scTRAIL, and VHH-D9-scTRAIL retain high affinity for the antigen and are selective between Kv10.1 and its closest relative, Kv10.2 (Ludwig et al., 2000), indicating that the furoin to the cargo did not alter the binding activity of the antibody moiety. The apparent affinity of VHH-D9-scTRAIL, in the pM range as determined by ELISA, was much higher than that of the VHH before fusion (nM). This could be due to the different approaches used to detect binding. For the VHH alone, ELISA detection used the His-tag from the vector for phage display. The tag was removed to generate the fusion construct, and therefore we needed to use an anti-TRAIL ELISA for detection of VHH-D9-scTRAIL. The presence of three TRAIL subunits per molecule of VHH could be responsible for the apparently higher affinity.

The efficacy of apoptosis induction of the new constructs was tested *in vitro* in DU-145 cells, a human prostate cancer cell line that had served already as a model for scFv62-TRAIL, a construct using monomeric TRAIL (Hartung et al., 2011; Hartung and Pardo, 2016). In this study, DU-145 cells also showed to be highly sensitive to the scTRAIL-based fusion, with and EC50 in the low nanomolar range. The effect of VHH-D9-scTRAIL required higher dose despite its higher affinity for the antigen (EC50 in the tens of nM), but the onset of the effect was faster and apoptosis induction was more intense than that of scFv62-scTRAIL. In spheroid experiments, however, both constructs were similarly efficacious in terms of growth inhibition. We did observe very intense apoptosis in DU-145 spheroids treated with both constructs, but the setup of the experiment makes quantification difficult. DU-145 spheroids are very compact, and do not change morphology upon treatment. Experiments using, e.g., two-photon microscopy will be needed for a more accurate quantification of apoptosis induction. Another factor that could influence the outcome is the fast growth of DU-145 cells (doubling time of approximately 29 h), which doubled the diameter of the spheroid in approximately 72 h.

In the human pancreatic cancer derived Capan-1, characterized by a much slower growth (doubling time of approximately 60–80 h), both fusion proteins induced intense apoptosis in standard 2D culture, with VHH-D9-scTRAIL being more potent than scFv62-scTRAIL. Tumor spheroids formed by Capan-1 cells alone are less compact than DU-145 spheroids, but compact spheroids were formed in the presence of RLT-PSC stellate stromal cells. Under both conditions, single culture or co-culture, both fusion constructs induced strong apoptosis also in this cell model. Clear apoptosis was detectable as early as 12 h after the start of treatment when using 3 ng/ml of the constructs, earlier in the case of VHH-D9-scTRAIL. The lower concentration used, 300 pg/ml, was also efficacious in the case of VHH-D9-scTRAIL, while the effect was less clear for scFv62-scTRAIL.

In summary, we generated in this study novel nanobody-based antibody-scTRAIL fusion constructs, whose binding unit shows high affinity for Kv10.1 and that are able to efficiently induce apoptosis of cancer cells. VHH-D9-scTRAIL was consistently faster and more potent than the scFv version.

Further investigations and *in vivo* studies are necessary to characterize other properties of VHH-D9-scTRAIL. The small size and remarkable stability of nanobodies as compared to other antibody forms should be advantageous in the therapeutic setting, but pharmacokinetic studies including tissue penetration, biodistribution, stability, and blood clearance will be required to confirm this assumption. Importantly, also for the potential use of the nanobody for diagnostic applications, it remains to be demonstrated that the increased efficacy of VHH-D9-scTRAIL is due to its high affinity for the antigen. It is still possible that the nanobody fusion induces a conformational change in scTRAIL that results in more efficient apoptosis induction, a possibility that merits careful consideration. The antigenic region is virtually identical in all Kv10.1 mammalian orthologs. This level of conservation will be important to predict potential undesired effects in preclinical models.

## Material and Methods

### Immunization and Phage Display

An adult male llama (*Lama glama*) was immunized subcutaneously at days 1, 20, 41, and 65 with 100 µg of the h1x antigen (E3-S5-pore segment fused to the C-terminal coiled coil domain of Kv10.1 and to thioredoxin). A phage display library was built as previously described (Behar et al., 2009). Briefly, the genes of VHH were amplified by RT-PCR using total RNA extracted from purified peripheral blood mononuclear cell and cloned into a phagemid vector to generate library of 1.3×10^7^ transformants. The library was created by the Nanobody platform of CRCM (Cancer Research Center of Marseille).

Phage display was performed as described (Even-Desrumeaux and Chames, 2012). For depletion against TRX, selection was performed using immobilized TRX or h1x on magnetic M450 Epoxy beads (Life Technologies). Both beads and phages were blocked with 20% BSA in PBS for 1 h at 4°C, and then incubated with the immobilized TRX (2 h, 4°C). The supernatant of the TRX slurry was then incubated on immobilized h1X for selection. The beads were washed 9 times with PBS plus 0.1% Tween 20 and twice with PBS and treated with 1 mg/ml trypsin for 30 min to elute the phages. 186 colonies of bacteria (*E. coli* TG1_TR_) infected with the output phages (3 sterile controls) were picked, grown overnight and transferred to round bottom 96 well plates and induced with Isopropyl β-D-1-thiogalactopyranoside (IPTG) overnight. The culture supernatants were used for screening by the enzyme linked immunosorbent assay (ELISA).

### scFv62-scTRAIL and VHH-D9-scTRAIL Constructs

Design and construction of the mouse-derived scFv has been described earlier (Hartung et al., 2011). The nanobodies against Kv10.1. The scTRAIL sequence was cloned together with scFv62, or VHH D9 into the multiple cloning site of the pSecTag2A plasmid. The fusion protein was expressed without the tags encoded in the pSecTag2A plasmid, which were eliminated by mutagenesis. The pSecTag2A protein expression vector with the corresponding insets was transfected into CHO-K1 cells. The plasmid carries the murine *kappa* light-chain leader peptide upstream of the multiple cloning site, and therefore directs the produced fusion protein through the ER and Golgi, resulting in excretion to the culture supernatant. Single clones were isolated from the transfected CHO-K1 cells and selected for those that showed the highest levels of secreted Ab-scTRAIL into the medium. Transfected cells were selected with Zeocin (3µg/ml in culture medium). For overexpression, the cells were cultured in a protein- and serum-free CHO medium [Panserin C6000 (PAN Biotech)] for five days at 30°C. The presence of the desired construct in the culture supernatant was confirmed by Western blot. No signal from other proteins was detected after electrophoresis and Comassie blue staining, and the supernatants were used without further purification. The product concentration was determined using the Human Quantikine TRAIL ELISA kit (R&D Systems) according to the manufacturer protocol against immobilized h1x. The expression yields ranged from 200-300 µg/L of culture.

### ELISA

96-well plates were coated overnight with 500 ng of h1x, h2x or TRX in 100 μl TBS per well in a wet chamber at room temperature. To block unspecific binding, wells were blocked with 2% (VHH ELISA) or 3% BSA (TRAIL ELISA) in TBS or PBS for 1 h. The VHHs (*E. coli* culture supernatants) or Ab-scTRAIL constructs were incubated at different concentration in 200 μl TBS for 2 h with shaking, and washed three times. VHH were detected by mouse anti-His antibody (Millipore) and a secondary anti-mouse, peroxidase conjugate (GE Healthcare). TRAIL signals were detected using anti-TRAIL conjugate and the detection buffer (R&D Systems, Quantikine TRAIL ELISA kit) according to the manufacturer’s protocol.

### Cell Culture

DU-145 and Capan-1, were purchased from ATCC, and CHO-K1 from DSMZ. Capan-1 cells were cultured in RPMI 1640 with 10%FCS; DU-145 in DMEM with 10% FCS and CHO-K1 cells in Ham’s F-12 medium with 10% FCS at 37°C in humidified 5% CO_2_ atmosphere. All media were purchased from Gibco Thermo Fisher Scientific. Transfection of Ab-scTRAIL in CHO-K1 cells was done with Lipofectamine 3000 (Thermo Fisher Scientific) as recommended by the manufacturer.

Spheroids were cultured in round bottom ultra-low attachment 96-well plates (Corning) at a density of 5,000 cells/well or 4,000 Capan-1 and 4,000 RLT-PSC cells per well in 2% Matrigel (Corning) and centrifuged at 1000x*g* for 10 min. Spheroid formation was monitored continuously in the IncuCyte system and treatments were added once spheroids were formed.

### Apoptosis Measurement

Cells or spheroids seeded in 96-well plates were monitored using the IncuCyte live-cell imaging system Essen Biosciences). Cells and spheroids were treated with the Ab-scTRAIL constructs in the presence of 8.9 µM cycloheximide (CHX) and Caspase 3/7 reagent (Essen Biosciences, red or green as required; 1:1,000 as recommended by the manufacturer). Caspase 3/7 reagent substrate crosses the cell membrane and its cleavage by activated caspase-3/7 results in the release of a DNA dye and fluorescent staining of nuclear DNA.

### Statistical Analysis

Data were analyzed using GraphPad Prism v.8 and are represented as mean ± SD. At least two biological replicates were performed for each analysis. Monolayer cell culture experiments were performed in triplicates, with two images per well and time point. Spheroid experiments were imaged also in triplicate wells, with a single image per well (covering the whole spheroid) at each time point.

## Data Availability Statement

The datasets generated for this study are available on request to the corresponding author.

## Author Contributions

FH, TK, PC, FA, and LP conceived and designed the study. KP and RK provided materials. FH, TK, and XS performed experiments, FH, TK, XS, PC, FA, and LP participated in data analysis and interpretation. All authors contributed to manuscript revision, read, and approved the submitted version.

## Funding

This project has received funding from the Max-Planck Society and from the European Union through Horizon 2020 research and innovation programme under the Marie Skłodowska-Curie grant agreement No. 813834-PHIONIC-H2020-MSCA-ITN-2018, and through the FP7 NMP “Large” Project NAMDIATREAM (contract no.246479). Open access publication costs funded by the Max-Planck-Society.

## Conflict of Interest

The authors declare that the research was conducted in the absence of any commercial or financial relationships that could be construed as a potential conflict of interest.

## Supplementary Material

Supplementary Video S1-6 Coordinate files for scFv62 and VHHD9 models in PDB format.

Supplementary Figure 1 Overview of the antigen used. **(A)** Aminoacid sequence of h1x. The Thioredoxin tag, E3 and C-terminal assembly domains are indicated by bars. **(B)** Alignment of the relevant sequences of h1x and Kv10.1 from several mammalian species. E3 is conserved among all species shown, and only a single residue in bovine and rat Kv10.1, and two in mouse, differ from the human sequence in the C-terminal assembly domain. The numbering corresponds to Homo sapiens Kv10.1, isoform 1 (UniProt O95259-2). UniProt IDs are: Pan troglodytes, H2Q129; Bos taurus, O18965; Rattus norvegicus, Q63472; Mus musculus, Q60603.

Supplementary Figure 2 Western blot of anti-Kv10.1 lead candidates. Purity and size of selected anti-Kv10.1 nanobodies lead candidates were validated by Western blot. Nanobodies were expressed in E. coli TG1TR. Three μg of the purified nanobodies were separated by SDS-PAGE. The blotted proteins were revealed with a HRP conjugated anti-His antibody. The nanobodies were expressed as monomers with a molecular weight ranging from 14 - 17 kDa (arrow). The higher molecular weight bands (bracket) correspond to the fusion between the nanobody and the p3 protein from the phage M13, as well as some degradation products from this p3.
