# Supplementary material for: A Novel Anti-Kv10.1 Nanobody Fused to Single-Chain TRAIL Enhances Apoptosis Induction in Cancer Cells
Source: Front Pharmacol. 2020 May 14;11:686. doi: 10.3389/fphar.2020.00686 (PMC7246340; doi:10.3389/fphar.2020.00686)
Supplement: Supplementary file 2 [file Table_1.docx]

Supplementary Material

## Supplementary Figures

**
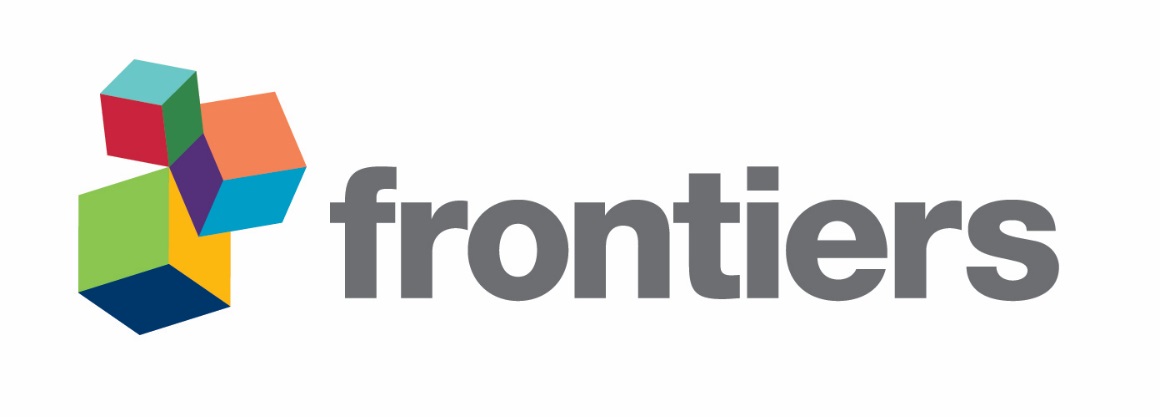
**

**Supplementary Figure 1.** **Western blot of anti-Kv10.1 lead candidates.**

Purity and size of selected anti-Kv10.1 nanobodies lead candidates were validated by Western blot. Nanobodies were expressed in E. coli TG1TR. Three μg of the purified nanobodies were separated by SDS-PAGE. The blotted proteins were revealed with a HRP conjugated anti-His antibody. The nanobodies were expressed as monomers with a molecular weight ranging from 14 - 17 kDa (arrow). The higher molecular weight bands (bracket) correspond to the fusion between the nanobody and the p3 protein from the phage M13, as well as some degradation products from this p3.

**Supplementary Figure 2. Overview of the antigen used. A.** Aminoacid sequence of h1x. The Thioredoxin tag, E3 and C-terminal assembly domains are indicated by bars. **B.** Alignment of the relevant sequences of h1x and Kv10.1 from several mammalian species. E3 is conserved among all species shown, and only a single residue in bovine and rat Kv10.1, and two in mouse, differ from the human sequence in the C-terminal assembly domain. The numbering corresponds to *Homo sapiens* Kv10.1, isoform 1 (UniProt O95259-2). UniProt IDs are: *Pan troglodytes,* H2Q129; *Bos taurus,* O18965; *Rattus norvegicus,* Q63472; *Mus musculus*, Q60603.

**Supplementary video 1.** Capan-1 spheroid, imaged for 24h after onset of treatment. Green fluorescence reports apoptosis.

**Supplementary video 2.** Capan-1 spheroid, treated with 3 ng/mL scFv62-scTRAIL and imaged for 24h after onset of treatment. Green fluorescence reports apoptosis.

**Supplementary video 3.** Capan-1 spheroid, treated with 3 ng/mL VHH-D9-scTRAIL and imaged for 24h after onset of treatment. Green fluorescence reports apoptosis.

**Supplementary video 4.** Capan-1/RLT-PSC stellate cells spheroid imaged for 24h after onset of treatment. Stellate cells are red fluorescent, and green fluorescence reports apoptosis.

**Supplementary video 5.** Capan-1/RLT-PSC stellate cells spheroid treated with 3 ng/mL scFv62-scTRAIL and imaged for 24h after onset of treatment. Stellate cells are red fluorescent, and green fluorescence reports apoptosis.

**Supplementary video 6.** Capan-1/RLT-PSC stellate cells spheroid treated with 3 ng/mL VHH-D9-scTRAIL and imaged for 24h after onset of treatment. Stellate cells are red fluorescent, and green fluorescence reports apoptosis.

**Supplementary Files.** Coordinates of VHH D9 and scFv62 used in Figure 3 in PDB format.

**Table I. Apparent affinity of the nanobodies determined by ELISA.**

| Nanobody | A9 | A12 | C4 | D9 | F5 | F6 | G1 | G4 |
| --- | --- | --- | --- | --- | --- | --- | --- | --- |
| K_d_ (nM) | 223 | 343 | 87 | 11 | 470 | 177 | 431 | 943 |
